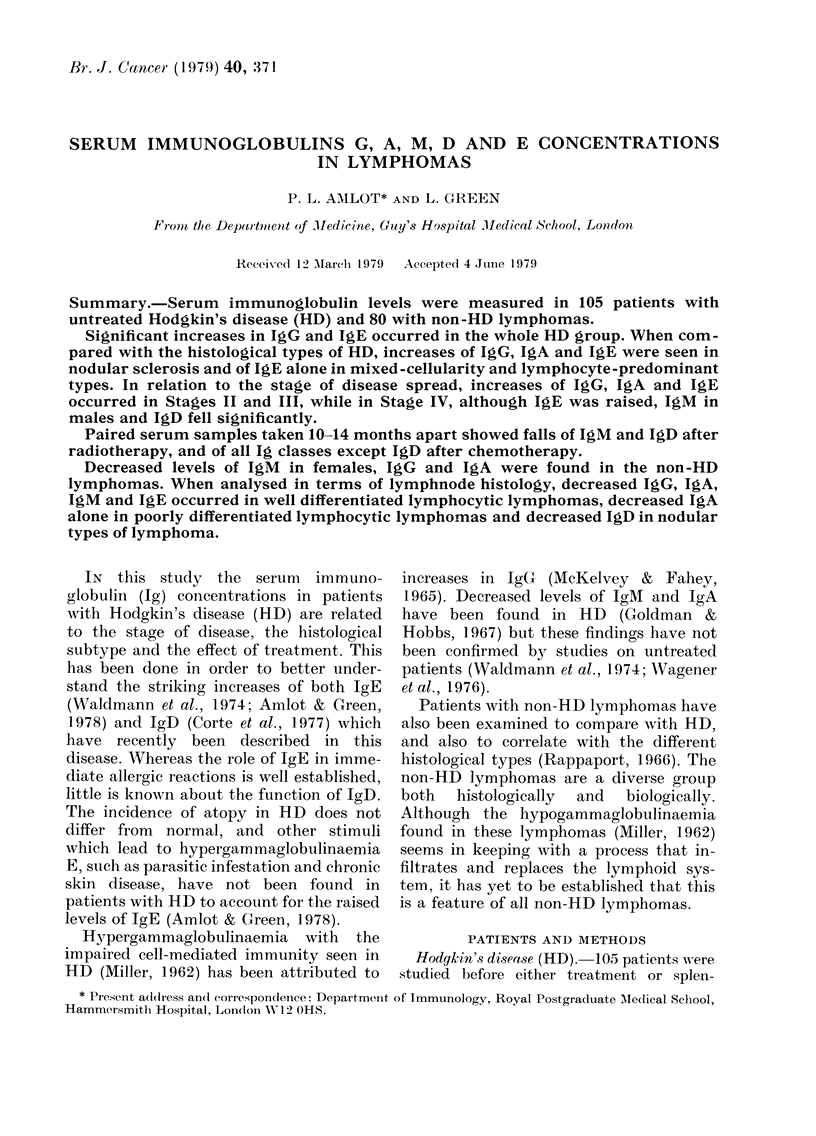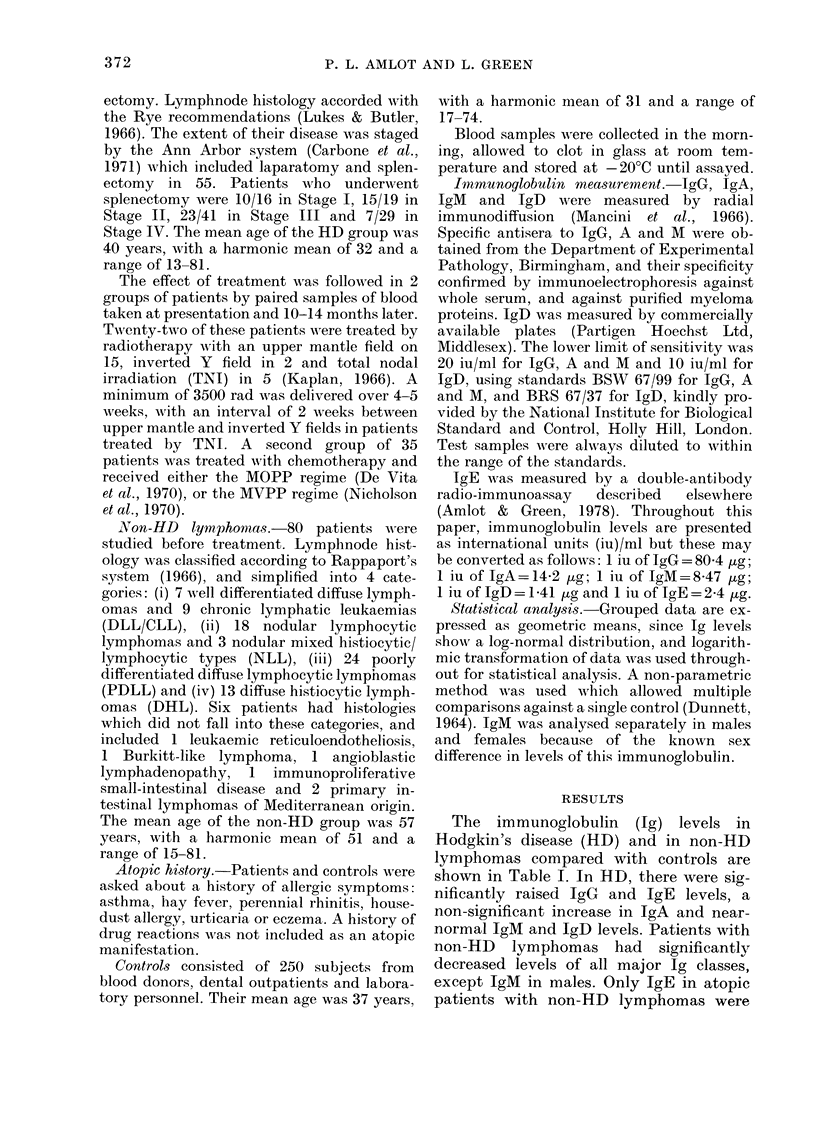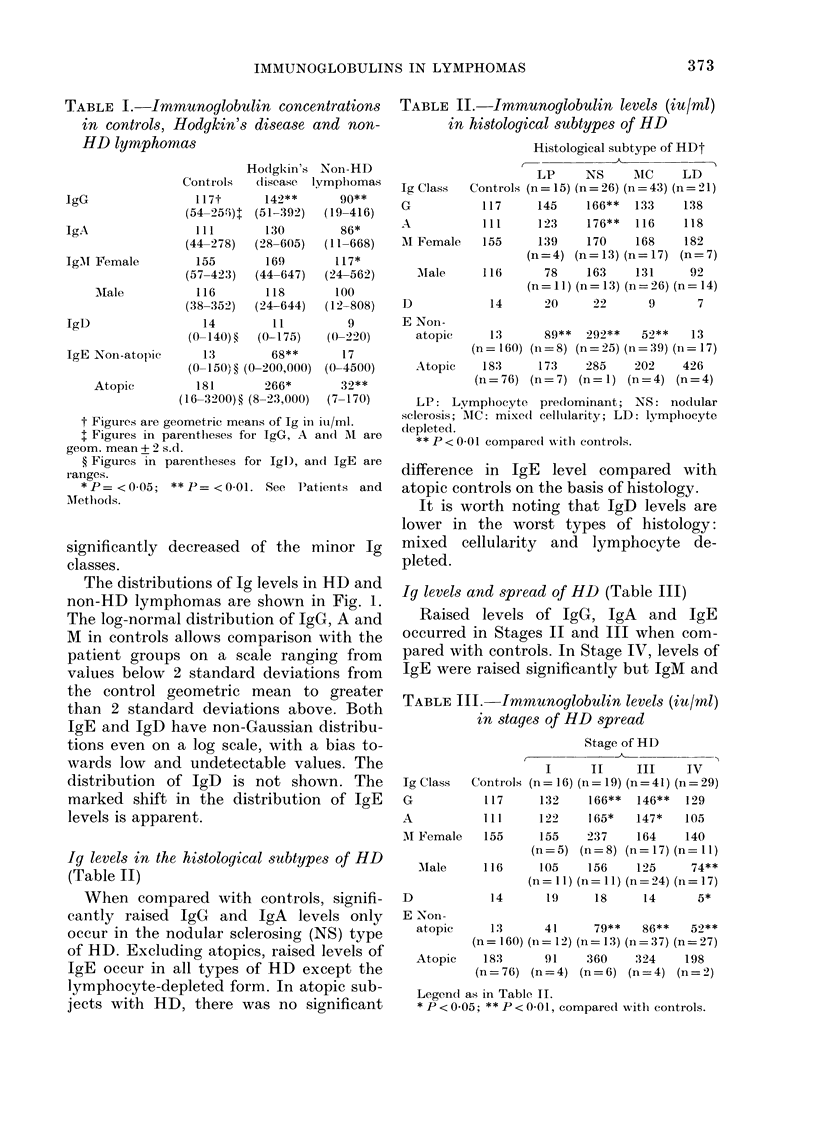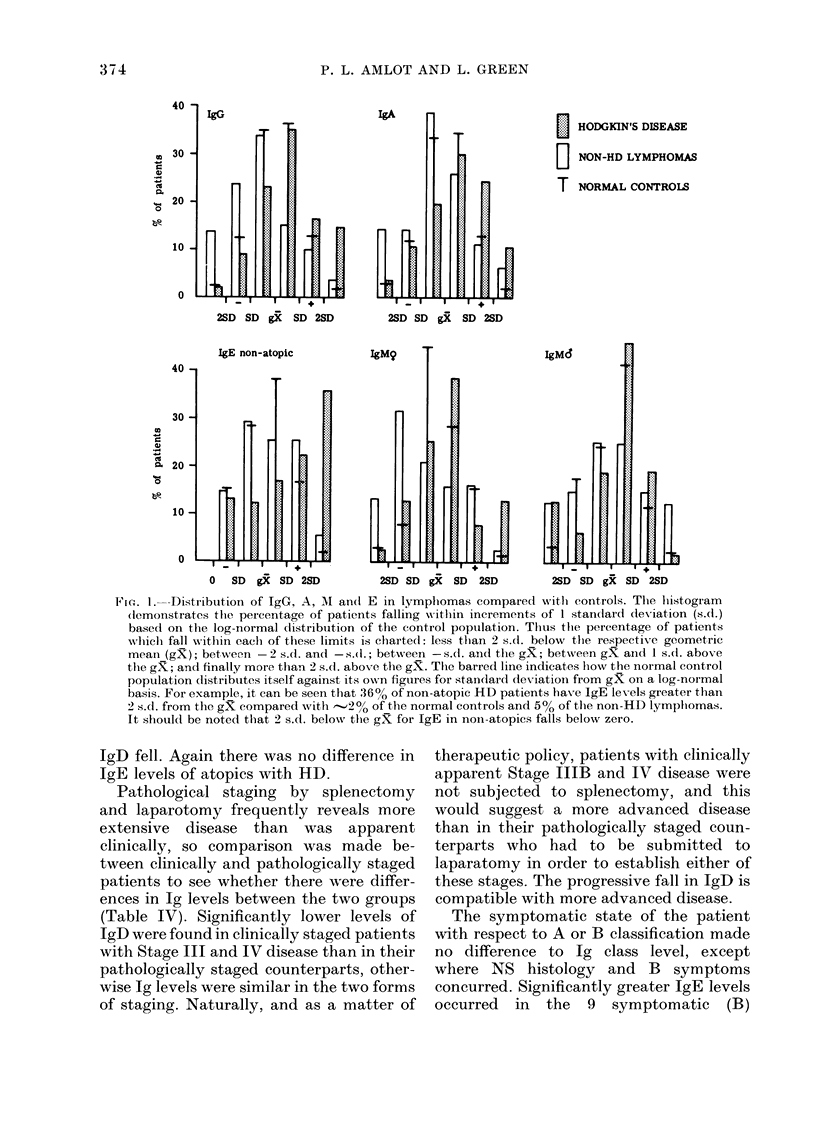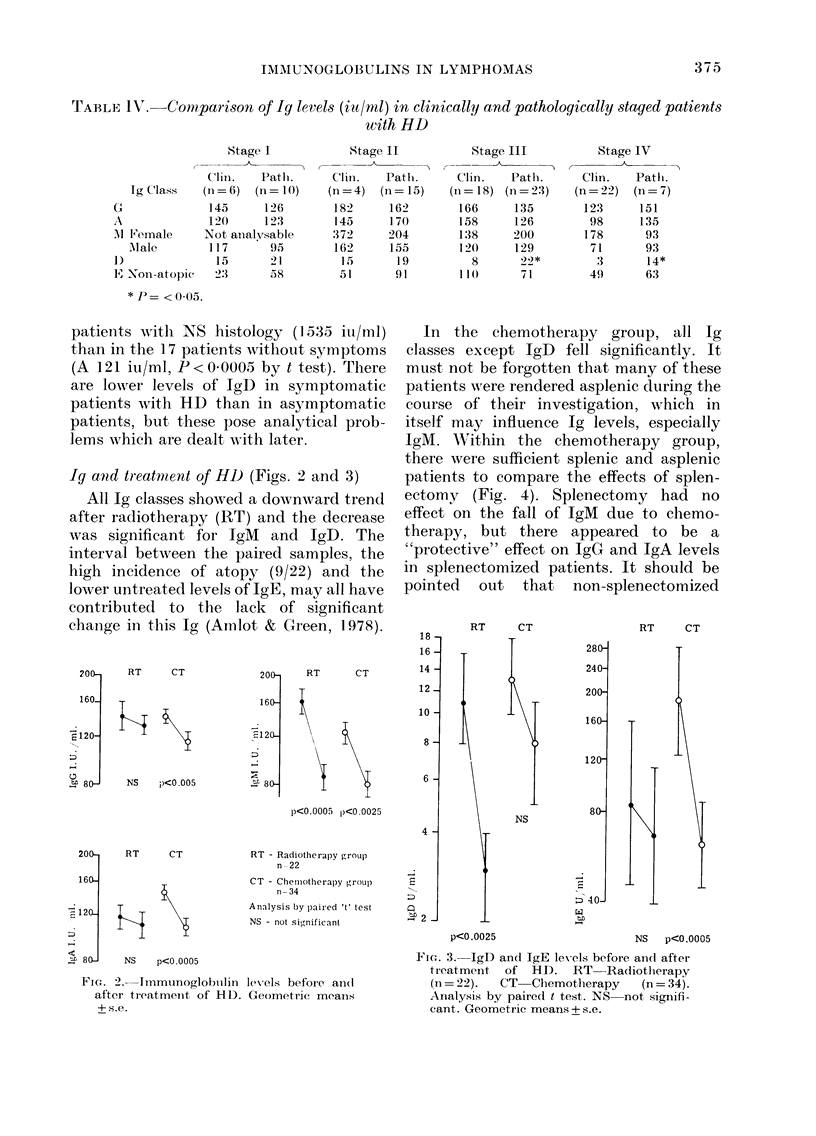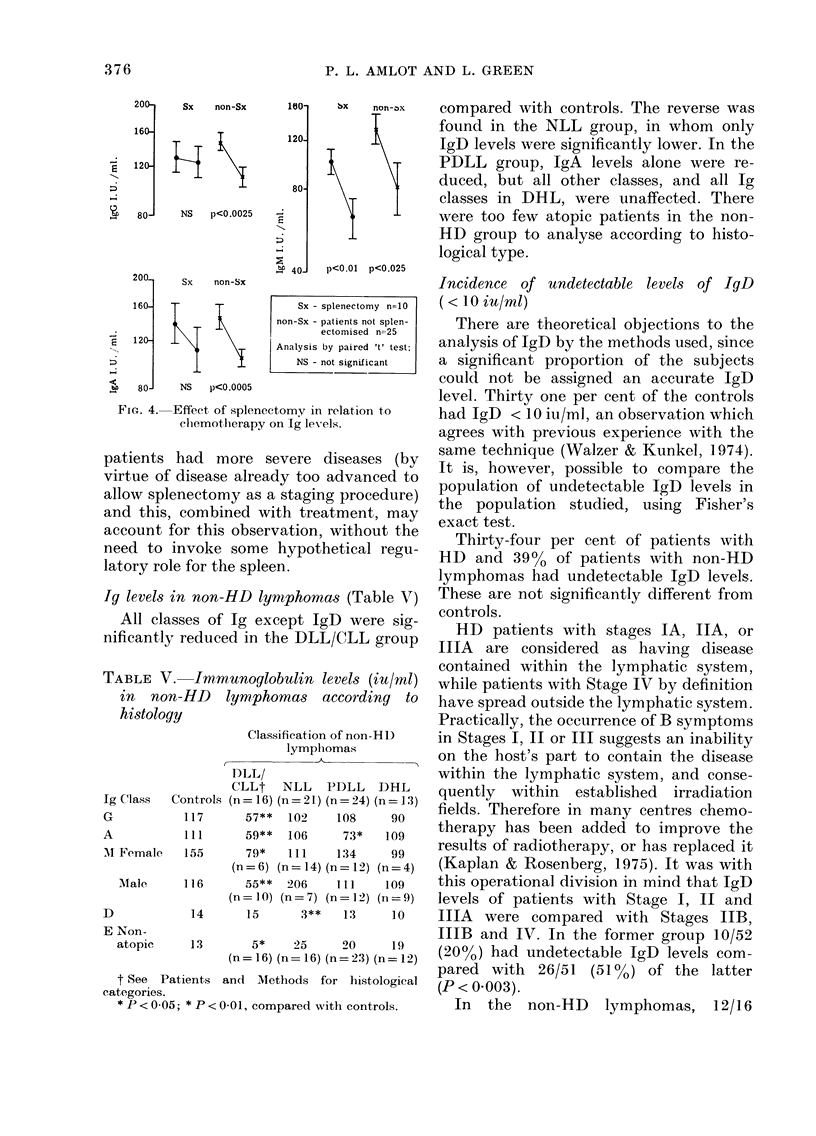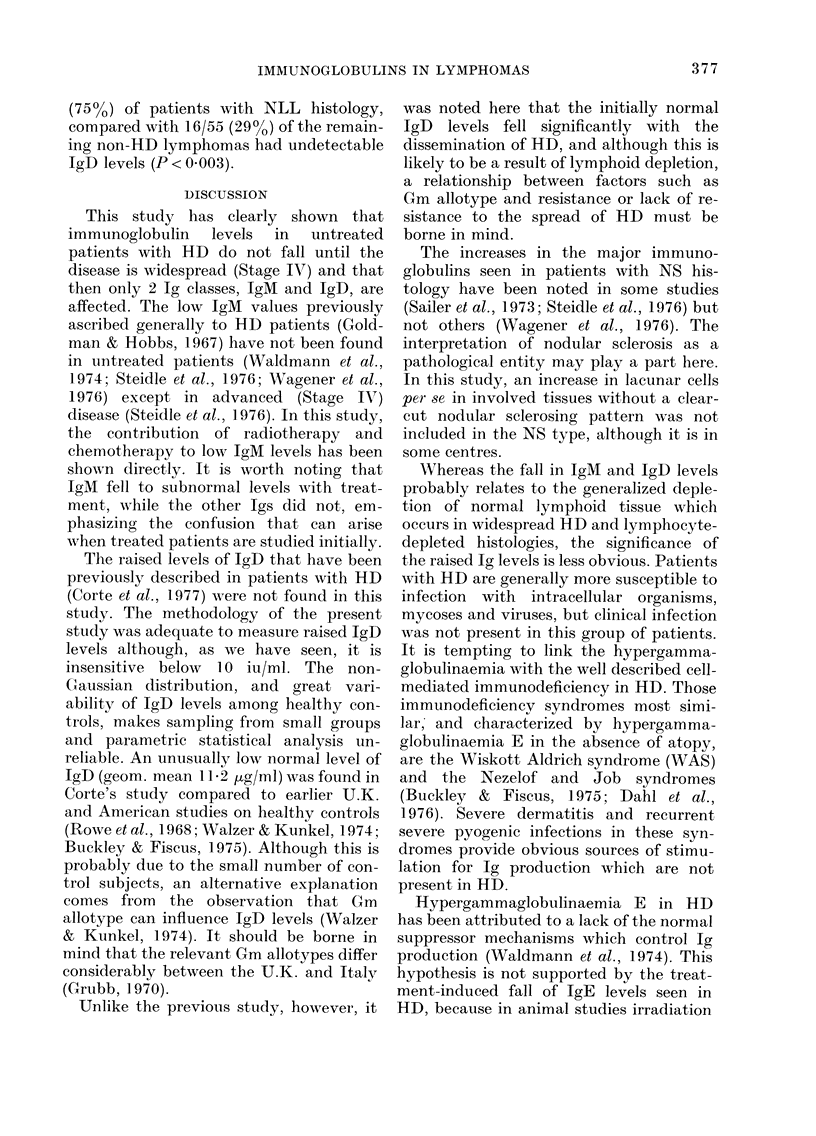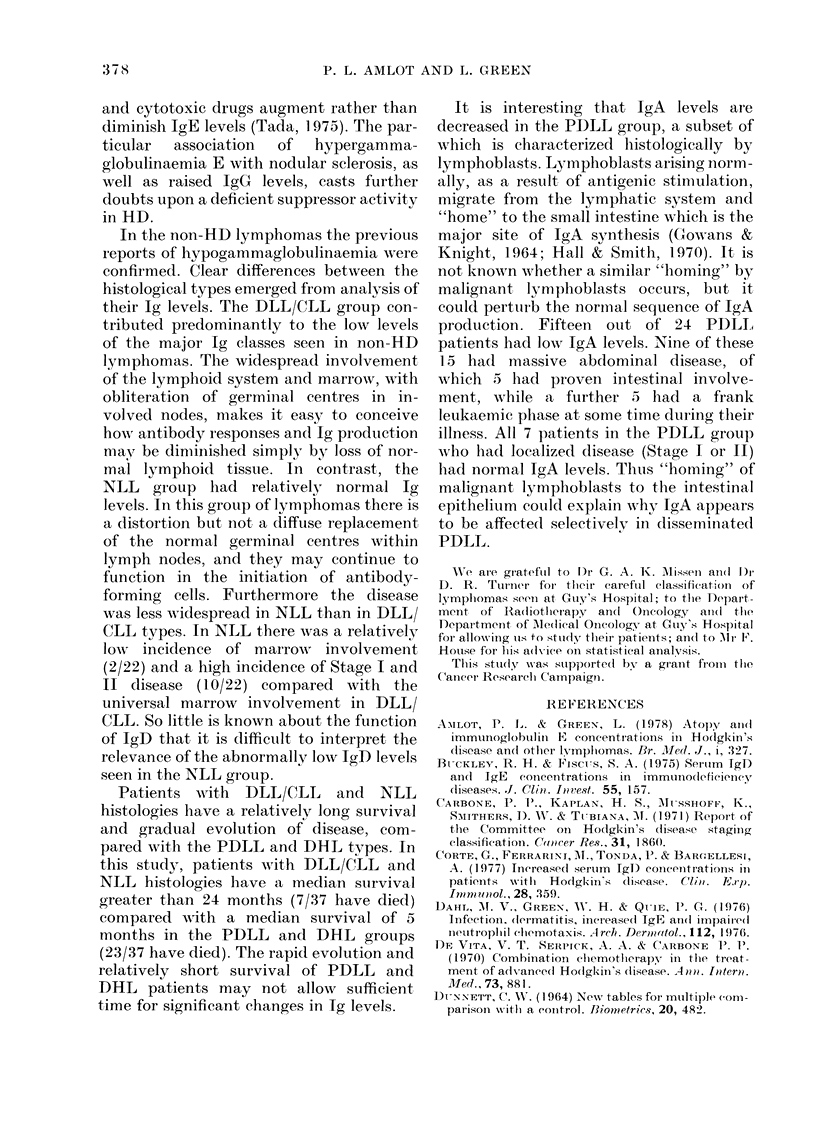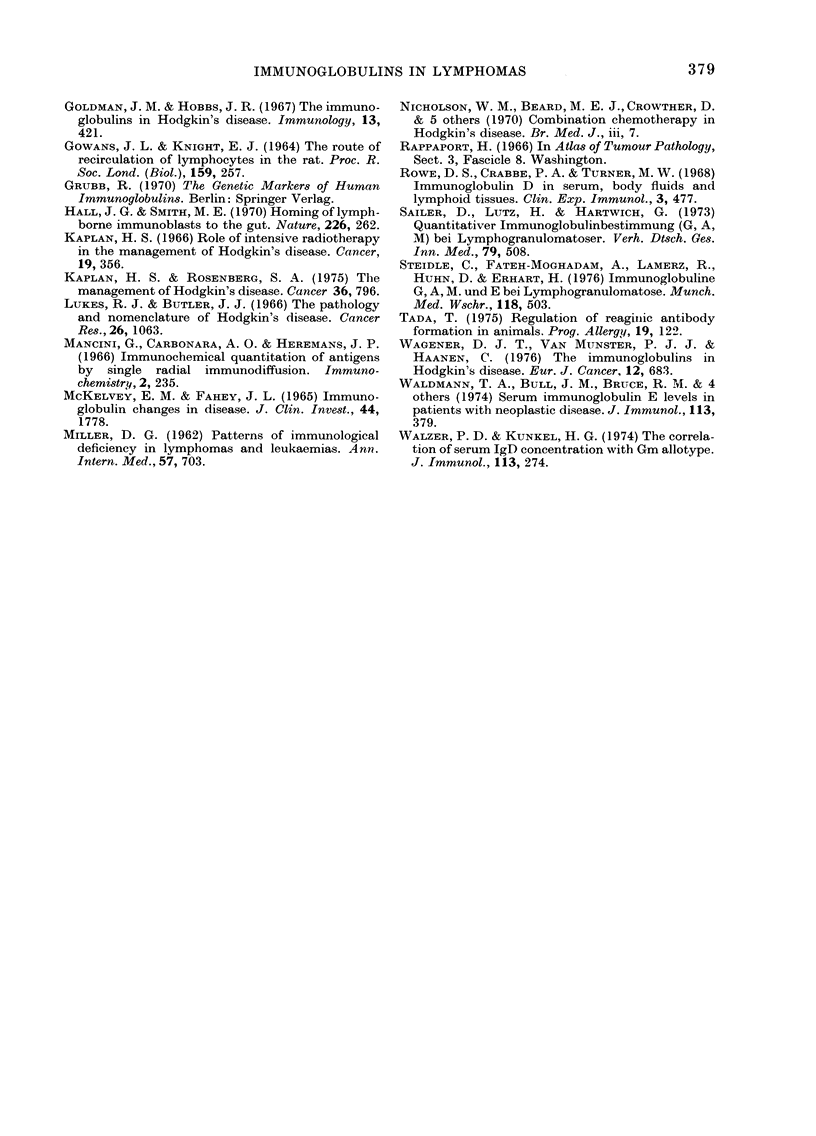# Serum immunoglobulins G, A, M, D and E concentrations in lymphomas.

**DOI:** 10.1038/bjc.1979.191

**Published:** 1979-09

**Authors:** P. L. Amlot, L. Green

## Abstract

Serum immunoglobulin levels were measured in 105 patients with untreated Hodgkin's disease (HD) and 80 with non-HD lymphomas. Significant increases in IgG and IgE occurred in the whole HD group. When compared with the histological types of HD, increases of IgG, IgA AND IgE were seen in nodular sclerosis and of IgE alone in mixed-cellularity and lymphocyte-predominant types. In relation to the stage of disease spread, increases of IgG, IgA AND IgE occurred in Stages II and III, while in Stage IV, although IgE was raised, IgM in males and IgD fell significantly. Paired serum samples taken 10-14 months apart showed falls of IgM and IgD after radiotherapy, and of all Ig classes except IgD after chemotherapy. Decreased levels of IgM in females, IgG and IgA were found in the non-HD lymphomas. When analysed in terms of lymphnode histology, decreased IgG, IgA, IgM and IgE occurred in well differentiated lymphocytic lymphomas, decreased IgA alone in poorly differentiated lymphocytic lymphomas and decreased IgD in nodular types of lymphoma.


*Br. J. Cancer* (1979) **40**, 371

### SERUM IMMUNOGLOBULINS G, A, M, D AND E CONCENTRATIONS IN LYMPHOMAS

P. L. AMLOT* AND L. GREEN

*From the Department of Medicine, Guy's Hospital Medical School, London*

Received 12 March 1979 Accepted 4 June 1979

**Summary.—Serum immunoglobulin levels were measured in 105 patients with untreated Hodgkin's disease (HD) and 80 with non-HD lymphomas.**

**Significant increases in IgG and IgE occurred in the whole HD group. When compared with the histological types of HD, increases of IgG, IgA and IgE were seen in nodular sclerosis and of IgE alone in mixed-cellularity and lymphocyte-predominant types. In relation to the stage of disease spread, increases of IgG, IgA and IgE occurred in Stages II and III, while in Stage IV, although IgE was raised, IgM in males and IgD fell significantly.**

**Paired serum samples taken 10–14 months apart showed falls of IgM and IgD after radiotherapy, and of all Ig classes except IgD after chemotherapy.**

**Decreased levels of IgM in females, IgG and IgA were found in the non-HD lymphomas. When analysed in terms of lymphnode histology, decreased IgG, IgA, IgM and IgE occurred in well differentiated lymphocytic lymphomas, decreased IgA alone in poorly differentiated lymphocytic lymphomas and decreased IgD in nodular types of lymphoma.**

In this study the serum immunoglobulin (Ig) concentrations in patients with Hodgkin's disease (HD) are related to the stage of disease, the histological subtype and the effect of treatment. This has been done in order to better understand the striking increases of both IgE (Waldmann *et al.*, 1974; Amlot & Green, 1978) and IgD (Corte *et al.*, 1977) which have recently been described in this disease. Whereas the role of IgE in immediate allergic reactions is well established, little is known about the function of IgD. The incidence of atopy in HD does not differ from normal, and other stimuli which lead to hypergammaglobulinaemia E, such as parasitic infestation and chronic skin disease, have not been found in patients with HD to account for the raised levels of IgE (Amlot & Green, 1978).

Hypergammaglobulinaemia with the impaired cell-mediated immunity seen in HD (Miller, 1962) has been attributed to increases in IgG (McKelvey & Fahey, 1965). Decreased levels of IgM and IgA have been found in HD (Goldman & Hobbs, 1967) but these findings have not been confirmed by studies on untreated patients (Waldmann *et al.*, 1974; Wagener *et al.*, 1976).

Patients with non-HD lymphomas have also been examined to compare with HD, and also to correlate with the different histological types (Rappaport, 1966). The non-HD lymphomas are a diverse group both histologically and biologically. Although the hypogammaglobulinaemia found in these lymphomas (Miller, 1962) seems in keeping with a process that infiltrates and replaces the lymphoid system, it has yet to be established that this is a feature of all non-HD lymphomas.

### PATIENTS AND METHODS

*Hodgkin's disease* (HD).—105 patients were studied before either treatment or splen-

* Present address and correspondence: Department of Immunology, Royal Postgraduate Medical School, Hammersmith Hospital, London W12 0HS.

ectomy. Lymphnode histology accorded with the Rye recommendations (Lukes & Butler, 1966). The extent of their disease was staged by the Ann Arbor system (Carbone *et al.*, 1971) which included laparatomy and splenectomy in 55. Patients who underwent splenectomy were 10/16 in Stage I, 15/19 in Stage II, 23/41 in Stage III and 7/29 in Stage IV. The mean age of the HD group was 40 years, with a harmonic mean of 32 and a range of 13–81.

The effect of treatment was followed in 2 groups of patients by paired samples of blood taken at presentation and 10–14 months later. Twenty-two of these patients were treated by radiotherapy with an upper mantle field on 15, inverted Y field in 2 and total nodal irradiation (TNI) in 5 (Kaplan, 1966). A minimum of 3500 rad was delivered over 4–5 weeks, with an interval of 2 weeks between upper mantle and inverted Y fields in patients treated by TNI. A second group of 35 patients was treated with chemotherapy and received either the MOPP regime (De Vita *et al.*, 1970), or the MVPP regime (Nicholson *et al.*, 1970).

*Non-HD lymphomas.*—80 patients were studied before treatment. Lymphnode histology was classified according to Rappaport's system (1966), and simplified into 4 categories: (i) 7 well differentiated diffuse lymphomas and 9 chronic lymphatic leukaemias (DLL/CLL), (ii) 18 nodular lymphocytic lymphomas and 3 nodular mixed histiocytic/lymphocytic types (NLL), (iii) 24 poorly differentiated diffuse lymphocytic lymphomas (PDLL) and (iv) 13 diffuse histiocytic lymphomas (DHL). Six patients had histologies which did not fall into these categories, and included 1 leukaemic reticuloendotheliosis, 1 Burkitt-like lymphoma, 1 angioblastic lymphadenopathy, 1 immunoproliferative small-intestinal disease and 2 primary intestinal lymphomas of Mediterranean origin. The mean age of the non-HD group was 57 years, with a harmonic mean of 51 and a range of 15–81.

*Atopic history.*—Patients and controls were asked about a history of allergic symptoms: asthma, hay fever, perennial rhinitis, housedust allergy, urticaria or eczema. A history of drug reactions was not included as an atopic manifestation.

*Controls* consisted of 250 subjects from blood donors, dental outpatients and laboratory personnel. Their mean age was 37 years, with a harmonic mean of 31 and a range of 17–74.

Blood samples were collected in the morning, allowed to clot in glass at room temperature and stored at −20°C until assayed.

*Immunoglobulin measurement.*—IgG, IgA, IgM and IgD were measured by radial immunodiffusion (Mancini *et al.*, 1966). Specific antisera to IgG, A and M were obtained from the Department of Experimental Pathology, Birmingham, and their specificity confirmed by immunoelectrophoresis against whole serum, and against purified myeloma proteins. IgD was measured by commercially available plates (Partigen Hoechst Ltd, Middlesex). The lower limit of sensitivity was 20 iu/ml for IgG, A and M and 10 iu/ml for IgD, using standards BSW 67/99 for IgG, A and M, and BRS 67/37 for IgD, kindly provided by the National Institute for Biological Standard and Control, Holly Hill, London. Test samples were always diluted to within the range of the standards.

IgE was measured by a double-antibody radio-immunoassay described elsewhere (Amlot & Green, 1978). Throughout this paper, immunoglobulin levels are presented as international units (iu)/ml but these may be converted as follows: 1 iu of IgG = 80·4 μg; 1 iu of IgA = 14·2 μg; 1 iu of IgM = 8·47 μg; 1 iu of IgD = 1·41 μg and 1 iu of IgE = 2·4 μg.

*Statistical analysis.*—Grouped data are expressed as geometric means, since Ig levels show a log-normal distribution, and logarithmic transformation of data was used throughout for statistical analysis. A non-parametric method was used which allowed multiple comparisons against a single control (Dunnett, 1964). IgM was analysed separately in males and females because of the known sex difference in levels of this immunoglobulin.

### RESULTS

The immunoglobulin (Ig) levels in Hodgkin's disease (HD) and in non-HD lymphomas compared with controls are shown in Table I. In HD, there were significantly raised IgG and IgE levels, a non-significant increase in IgA and near-normal IgM and IgD levels. Patients with non-HD lymphomas had significantly decreased levels of all major Ig classes, except IgM in males. Only IgE in atopic patients with non-HD lymphomas were

TABLE I.—*Immunoglobulin concentrations in controls, Hodgkin's disease and non-HD lymphomas*

| | Controls | Hodgkin's disease | Non-HD lymphomas |
|---|---|---|---|
| IgG | 117† (54–256)‡ | 142** (51–392) | 90** (19–416) |
| IgA | 111 (44–278) | 130 (28–605) | 86* (11–668) |
| IgM Female | 155 (57–423) | 169 (44–647) | 117* (24–562) |
| Male | 116 (38–352) | 118 (24–644) | 100 (12–808) |
| IgD | 14 (0–140)§ | 11 (0–175) | 9 (0–220) |
| IgE Non-atopic | 13 (0–150)§ | 68** (0–200,000) | 17 (0–4500) |
| Atopic | 181 (16–3200)§ | 266* (8–23,000) | 32** (7–170) |

† Figures are geometric means of Ig in iu/ml.

‡ Figures in parentheses for IgG, A and M are geom. mean ± 2 s.d.

§ Figures in parentheses for IgD, and IgE are ranges.

* $P = < 0{\cdot}05$; ** $P = < 0{\cdot}01$. See Patients and Methods.

significantly decreased of the minor Ig classes.

The distributions of Ig levels in HD and non-HD lymphomas are shown in Fig. 1. The log-normal distribution of IgG, A and M in controls allows comparison with the patient groups on a scale ranging from values below 2 standard deviations from the control geometric mean to greater than 2 standard deviations above. Both IgE and IgD have non-Gaussian distributions even on a log scale, with a bias towards low and undetectable values. The distribution of IgD is not shown. The marked shift in the distribution of IgE levels is apparent.

*Ig levels in the histological subtypes of HD* (Table II)

When compared with controls, significantly raised IgG and IgA levels only occur in the nodular sclerosing (NS) type of HD. Excluding atopics, raised levels of IgE occur in all types of HD except the lymphocyte-depleted form. In atopic subjects with HD, there was no significant

TABLE II.—*Immunoglobulin levels (iu/ml) in histological subtypes of HD*

| | | Histological subtype of HD† | | | |
|---|---|---|---|---|---|
| Ig Class | Controls | LP (n = 15) | NS (n = 26) | MC (n = 43) | LD (n = 21) |
| G | 117 | 145 | 166** | 133 | 138 |
| A | 111 | 123 | 176** | 116 | 118 |
| M Female | 155 | 139 (n = 4) | 170 (n = 13) | 168 (n = 17) | 182 (n = 7) |
| Male | 116 | 78 (n = 11) | 163 (n = 13) | 131 (n = 26) | 92 (n = 14) |
| D | 14 | 20 | 22 | 9 | 7 |
| E Non-atopic | 13 (n = 160) | 89** (n = 8) | 292** (n = 25) | 52** (n = 39) | 13 (n = 17) |
| Atopic | 183 (n = 76) | 173 (n = 7) | 285 (n = 1) | 202 (n = 4) | 426 (n = 4) |

LP: Lymphocyte predominant; NS: nodular sclerosis; MC: mixed cellularity; LD: lymphocyte depleted.
** $P < 0{\cdot}01$ compared with controls.

difference in IgE level compared with atopic controls on the basis of histology.

It is worth noting that IgD levels are lower in the worst types of histology: mixed cellularity and lymphocyte depleted.

*Ig levels and spread of HD* (Table III)

Raised levels of IgG, IgA and IgE occurred in Stages II and III when compared with controls. In Stage IV, levels of IgE were raised significantly but IgM and

TABLE III.—*Immunoglobulin levels (iu/ml) in stages of HD spread*

| | | Stage of HD | | | |
|---|---|---|---|---|---|
| Ig Class | Controls | I (n = 16) | II (n = 19) | III (n = 41) | IV (n = 29) |
| G | 117 | 132 | 166** | 146** | 129 |
| A | 111 | 122 | 165* | 147* | 105 |
| M Female | 155 | 155 (n = 5) | 237 (n = 8) | 164 (n = 17) | 140 (n = 11) |
| Male | 116 | 105 (n = 11) | 156 (n = 11) | 125 (n = 24) | 74** (n = 17) |
| D | 14 | 19 | 18 | 14 | 5* |
| E Non-atopic | 13 (n = 160) | 41 (n = 12) | 79** (n = 13) | 86** (n = 37) | 52** (n = 27) |
| Atopic | 183 (n = 76) | 91 (n = 4) | 360 (n = 6) | 324 (n = 4) | 198 (n = 2) |

Legend as in Table II.
* $P < 0{\cdot}05$; ** $P < 0{\cdot}01$, compared with controls.

FIG. 1.—Distribution of IgG, A, M and E in lymphomas compared with controls. The histogram demonstrates the percentage of patients falling within increments of 1 standard deviation (s.d.) based on the log-normal distribution of the control population. Thus the percentage of patients which fall within each of these limits is charted: less than 2 s.d. below the respective geometric mean (gX̄); between −2 s.d. and −s.d.; between −s.d. and the gX̄; between gX̄ and 1 s.d. above the gX̄; and finally more than 2 s.d. above the gX̄. The barred line indicates how the normal control population distributes itself against its own figures for standard deviation from gX̄ on a log-normal basis. For example, it can be seen that 36% of non-atopic HD patients have IgE levels greater than 2 s.d. from the gX̄ compared with ~2% of the normal controls and 5% of the non-HD lymphomas. It should be noted that 2 s.d. below the gX̄ for IgE in non-atopics falls below zero.

IgD fell. Again there was no difference in IgE levels of atopics with HD.

Pathological staging by splenectomy and laparotomy frequently reveals more extensive disease than was apparent clinically, so comparison was made between clinically and pathologically staged patients to see whether there were differences in Ig levels between the two groups (Table IV). Significantly lower levels of IgD were found in clinically staged patients with Stage III and IV disease than in their pathologically staged counterparts, otherwise Ig levels were similar in the two forms of staging. Naturally, and as a matter of therapeutic policy, patients with clinically apparent Stage IIIB and IV disease were not subjected to splenectomy, and this would suggest a more advanced disease than in their pathologically staged counterparts who had to be submitted to laparatomy in order to establish either of these stages. The progressive fall in IgD is compatible with more advanced disease.

The symptomatic state of the patient with respect to A or B classification made no difference to Ig class level, except where NS histology and B symptoms concurred. Significantly greater IgE levels occurred in the 9 symptomatic (B)

TABLE IV.—*Comparison of Ig levels (iu/ml) in clinically and pathologically staged patients with HD*

| | Stage I | | Stage II | | Stage III | | Stage IV | |
|---|---|---|---|---|---|---|---|---|
| Ig Class | Clin. (n = 6) | Path. (n = 10) | Clin. (n = 4) | Path. (n = 15) | Clin. (n = 18) | Path. (n = 23) | Clin. (n = 22) | Path. (n = 7) |
| G | 145 | 126 | 182 | 162 | 166 | 135 | 123 | 151 |
| A | 120 | 123 | 145 | 170 | 158 | 126 | 98 | 135 |
| M Female | Not analysable | | 372 | 204 | 138 | 200 | 178 | 93 |
| Male | 117 | 95 | 162 | 155 | 120 | 129 | 71 | 93 |
| D | 15 | 21 | 15 | 19 | 8 | 22* | 3 | 14* |
| E Non-atopic | 23 | 58 | 51 | 91 | 110 | 71 | 49 | 63 |

* $P = < 0{\cdot}05$.

patients with NS histology (1535 iu/ml) than in the 17 patients without symptoms (A 121 iu/ml, $P < 0{\cdot}0005$ by $t$ test). There are lower levels of IgD in symptomatic patients with HD than in asymptomatic patients, but these pose analytical problems which are dealt with later.

*Ig and treatment of HD* (Figs. 2 and 3)

All Ig classes showed a downward trend after radiotherapy (RT) and the decrease was significant for IgM and IgD. The interval between the paired samples, the high incidence of atopy (9/22) and the lower untreated levels of IgE, may all have contributed to the lack of significant change in this Ig (Amlot & Green, 1978).

In the chemotherapy group, all Ig classes except IgD fell significantly. It must not be forgotten that many of these patients were rendered asplenic during the course of their investigation, which in itself may influence Ig levels, especially IgM. Within the chemotherapy group, there were sufficient splenic and asplenic patients to compare the effects of splenectomy (Fig. 4). Splenectomy had no effect on the fall of IgM due to chemotherapy, but there appeared to be a "protective" effect on IgG and IgA levels in splenectomized patients. It should be pointed out that non-splenectomized

FIG. 2.—Immunoglobulin levels before and after treatment of HD. Geometric means ± s.e.

FIG. 3.—IgD and IgE levels before and after treatment of HD. RT—Radiotherapy (n = 22). CT—Chemotherapy (n = 34). Analysis by paired $t$ test. NS—not significant. Geometric means ± s.e.

FIG. 4.—Effect of splenectomy in relation to chemotherapy on Ig levels.

patients had more severe diseases (by virtue of disease already too advanced to allow splenectomy as a staging procedure) and this, combined with treatment, may account for this observation, without the need to invoke some hypothetical regulatory role for the spleen.

*Ig levels in non-HD lymphomas* (Table V)

All classes of Ig except IgD were significantly reduced in the DLL/CLL group

TABLE V.—*Immunoglobulin levels (iu/ml) in non-HD lymphomas according to histology*

| | | Classification of non-HD lymphomas | | | |
|---|---|---|---|---|---|
| Ig Class | Controls | DLL/ CLL† (n = 16) | NLL (n = 21) | PDLL (n = 24) | DHL (n = 13) |
| G | 117 | 57** | 102 | 108 | 90 |
| A | 111 | 59** | 106 | 73* | 109 |
| M Female | 155 | 79* (n = 6) | 111 (n = 14) | 134 (n = 12) | 99 (n = 4) |
| Male | 116 | 55** (n = 10) | 206 (n = 7) | 111 (n = 12) | 109 (n = 9) |
| D | 14 | 15 | 3** | 13 | 10 |
| E Non-atopic | 13 | 5* (n = 16) | 25 (n = 16) | 20 (n = 23) | 19 (n = 12) |

† See Patients and Methods for histological categories.
* $P < 0{\cdot}05$; * $P < 0{\cdot}01$, compared with controls.

compared with controls. The reverse was found in the NLL group, in whom only IgD levels were significantly lower. In the PDLL group, IgA levels alone were reduced, but all other classes, and all Ig classes in DHL, were unaffected. There were too few atopic patients in the non-HD group to analyse according to histological type.

*Incidence of undetectable levels of IgD (< 10 iu/ml)*

There are theoretical objections to the analysis of IgD by the methods used, since a significant proportion of the subjects could not be assigned an accurate IgD level. Thirty one per cent of the controls had IgD < 10 iu/ml, an observation which agrees with previous experience with the same technique (Walzer & Kunkel, 1974). It is, however, possible to compare the population of undetectable IgD levels in the population studied, using Fisher's exact test.

Thirty-four per cent of patients with HD and 39% of patients with non-HD lymphomas had undetectable IgD levels. These are not significantly different from controls.

HD patients with stages IA, IIA, or IIIA are considered as having disease contained within the lymphatic system, while patients with Stage IV by definition have spread outside the lymphatic system. Practically, the occurrence of B symptoms in Stages I, II or III suggests an inability on the host's part to contain the disease within the lymphatic system, and consequently within established irradiation fields. Therefore in many centres chemotherapy has been added to improve the results of radiotherapy, or has replaced it (Kaplan & Rosenberg, 1975). It was with this operational division in mind that IgD levels of patients with Stage I, II and IIIA were compared with Stages IIB, IIIB and IV. In the former group 10/52 (20%) had undetectable IgD levels compared with 26/51 (51%) of the latter ($P < 0{\cdot}003$).

In the non-HD lymphomas, 12/16

(75%) of patients with NLL histology, compared with 16/55 (29%) of the remaining non-HD lymphomas had undetectable IgD levels ($P < 0{\cdot}003$).

DISCUSSION

This study has clearly shown that immunoglobulin levels in untreated patients with HD do not fall until the disease is widespread (Stage IV) and that then only 2 Ig classes, IgM and IgD, are affected. The low IgM values previously ascribed generally to HD patients (Goldman & Hobbs, 1967) have not been found in untreated patients (Waldmann *et al.*, 1974; Steidle *et al.*, 1976; Wagener *et al.*, 1976) except in advanced (Stage IV) disease (Steidle *et al.*, 1976). In this study, the contribution of radiotherapy and chemotherapy to low IgM levels has been shown directly. It is worth noting that IgM fell to subnormal levels with treatment, while the other Igs did not, emphasizing the confusion that can arise when treated patients are studied initially.

The raised levels of IgD that have been previously described in patients with HD (Corte *et al.*, 1977) were not found in this study. The methodology of the present study was adequate to measure raised IgD levels although, as we have seen, it is insensitive below 10 iu/ml. The non-Gaussian distribution, and great variability of IgD levels among healthy controls, makes sampling from small groups and parametric statistical analysis unreliable. An unusually low normal level of IgD (geom. mean 11·2 $\mu$g/ml) was found in Corte's study compared to earlier U.K. and American studies on healthy controls (Rowe *et al.*, 1968; Walzer & Kunkel, 1974; Buckley & Fiscus, 1975). Although this is probably due to the small number of control subjects, an alternative explanation comes from the observation that Gm allotype can influence IgD levels (Walzer & Kunkel, 1974). It should be borne in mind that the relevant Gm allotypes differ considerably between the U.K. and Italy (Grubb, 1970).

Unlike the previous study, however, it was noted here that the initially normal IgD levels fell significantly with the dissemination of HD, and although this is likely to be a result of lymphoid depletion, a relationship between factors such as Gm allotype and resistance or lack of resistance to the spread of HD must be borne in mind.

The increases in the major immunoglobulins seen in patients with NS histology have been noted in some studies (Sailer *et al.*, 1973; Steidle *et al.*, 1976) but not others (Wagener *et al.*, 1976). The interpretation of nodular sclerosis as a pathological entity may play a part here. In this study, an increase in lacunar cells *per se* in involved tissues without a clear-cut nodular sclerosing pattern was not included in the NS type, although it is in some centres.

Whereas the fall in IgM and IgD levels probably relates to the generalized depletion of normal lymphoid tissue which occurs in widespread HD and lymphocyte-depleted histologies, the significance of the raised Ig levels is less obvious. Patients with HD are generally more susceptible to infection with intracellular organisms, mycoses and viruses, but clinical infection was not present in this group of patients. It is tempting to link the hypergammaglobulinaemia with the well described cell-mediated immunodeficiency in HD. Those immunodeficiency syndromes most similar, and characterized by hypergammaglobulinaemia E in the absence of atopy, are the Wiskott Aldrich syndrome (WAS) and the Nezelof and Job syndromes (Buckley & Fiscus, 1975; Dahl *et al.*, 1976). Severe dermatitis and recurrent severe pyogenic infections in these syndromes provide obvious sources of stimulation for Ig production which are not present in HD.

Hypergammaglobulinaemia E in HD has been attributed to a lack of the normal suppressor mechanisms which control Ig production (Waldmann *et al.*, 1974). This hypothesis is not supported by the treatment-induced fall of IgE levels seen in HD, because in animal studies irradiation and cytotoxic drugs augment rather than diminish IgE levels (Tada, 1975). The particular association of hypergammaglobulinaemia E with nodular sclerosis, as well as raised IgG levels, casts further doubts upon a deficient suppressor activity in HD.

In the non-HD lymphomas the previous reports of hypogammaglobulinaemia were confirmed. Clear differences between the histological types emerged from analysis of their Ig levels. The DLL/CLL group contributed predominantly to the low levels of the major Ig classes seen in non-HD lymphomas. The widespread involvement of the lymphoid system and marrow, with obliteration of germinal centres in involved nodes, makes it easy to conceive how antibody responses and Ig production may be diminished simply by loss of normal lymphoid tissue. In contrast, the NLL group had relatively normal Ig levels. In this group of lymphomas there is a distortion but not a diffuse replacement of the normal germinal centres within lymph nodes, and they may continue to function in the initiation of antibody-forming cells. Furthermore the disease was less widespread in NLL than in DLL/CLL types. In NLL there was a relatively low incidence of marrow involvement (2/22) and a high incidence of Stage I and II disease (10/22) compared with the universal marrow involvement in DLL/CLL. So little is known about the function of IgD that it is difficult to interpret the relevance of the abnormally low IgD levels seen in the NLL group.

Patients with DLL/CLL and NLL histologies have a relatively long survival and gradual evolution of disease, compared with the PDLL and DHL types. In this study, patients with DLL/CLL and NLL histologies have a median survival greater than 24 months (7/37 have died) compared with a median survival of 5 months in the PDLL and DHL groups (23/37 have died). The rapid evolution and relatively short survival of PDLL and DHL patients may not allow sufficient time for significant changes in Ig levels.

It is interesting that IgA levels are decreased in the PDLL group, a subset of which is characterized histologically by lymphoblasts. Lymphoblasts arising normally, as a result of antigenic stimulation, migrate from the lymphatic system and "home" to the small intestine which is the major site of IgA synthesis (Gowans & Knight, 1964; Hall & Smith, 1970). It is not known whether a similar "homing" by malignant lymphoblasts occurs, but it could perturb the normal sequence of IgA production. Fifteen out of 24 PDLL patients had low IgA levels. Nine of these 15 had massive abdominal disease, of which 5 had proven intestinal involvement, while a further 5 had a frank leukaemic phase at some time during their illness. All 7 patients in the PDLL group who had localized disease (Stage I or II) had normal IgA levels. Thus "homing" of malignant lymphoblasts to the intestinal epithelium could explain why IgA appears to be affected selectively in disseminated PDLL.

We are grateful to Dr G. A. K. Missen and Dr D. R. Turner for their careful classification of lymphomas seen at Guy's Hospital; to the Department of Radiotherapy and Oncology and the Department of Medical Oncology at Guy's Hospital for allowing us to study their patients; and to Mr F. House for his advice on statistical analysis.

This study was supported by a grant from the Cancer Research Campaign.

### REFERENCES

AMLOT, P. L. & GREEN, L. (1978) Atopy and immunoglobulin E concentrations in Hodgkin's disease and other lymphomas. *Br. Med. J.*, i, 327.

BUCKLEY, R. H. & FISCUS, S. A. (1975) Serum IgD and IgE concentrations in immunodeficiency diseases. *J. Clin. Invest.* **55,** 157.

CARBONE, P. P., KAPLAN, H. S., MUSSHOFF, K., SMITHERS, D. W. & TUBIANA, M. (1971) Report of the Committee on Hodgkin's disease staging classification. *Cancer Res.*, **31,** 1860.

CORTE, G., FERRARINI, M., TONDA, P. & BARGELLESI, A. (1977) Increased serum IgD concentrations in patients with Hodgkin's disease. *Clin. Exp. Immunol.*, **28,** 359.

DAHL, M. V., GREEN, W. H. & QUIE, P. G. (1976) Infection, dermatitis, increased IgE and impaired neutrophil chemotaxis. *Arch. Dermatol.*, **112,** 1976.

DE VITA, V. T. SERPICK, A. A. & CARBONE P. P. (1970) Combination chemotherapy in the treatment of advanced Hodgkin's disease. *Ann. Intern. Med.*, **73,** 881.

DUNNETT, C. W. (1964) New tables for multiple comparison with a control. *Biometrics*, **20,** 482.

GOLDMAN, J. M. & HOBBS, J. R. (1967) The immunoglobulins in Hodgkin's disease. *Immunology*, **13**, 421.

GOWANS, J. L. & KNIGHT, E. J. (1964) The route of recirculation of lymphocytes in the rat. *Proc. R. Soc. Lond.* (*Biol.*), **159**, 257.

GRUBB, R. (1970) *The Genetic Markers of Human Immunoglobulins*. Berlin: Springer Verlag.

HALL, J. G. & SMITH, M. E. (1970) Homing of lymph-borne immunoblasts to the gut. *Nature*, **226**, 262.

KAPLAN, H. S. (1966) Role of intensive radiotherapy in the management of Hodgkin's disease. *Cancer*, **19**, 356.

KAPLAN, H. S. & ROSENBERG, S. A. (1975) The management of Hodgkin's disease. *Cancer* **36**, 796.

LUKES, R. J. & BUTLER, J. J. (1966) The pathology and nomenclature of Hodgkin's disease. *Cancer Res.*, **26**, 1063.

MANCINI, G., CARBONARA, A. O. & HEREMANS, J. P. (1966) Immunochemical quantitation of antigens by single radial immunodiffusion. *Immunochemistry*, **2**, 235.

MCKELVEY, E. M. & FAHEY, J. L. (1965) Immunoglobulin changes in disease. *J. Clin. Invest.*, **44**, 1778.

MILLER, D. G. (1962) Patterns of immunological deficiency in lymphomas and leukaemias. *Ann. Intern. Med.*, **57**, 703.

NICHOLSON, W. M., BEARD, M. E. J., CROWTHER, D. & 5 others (1970) Combination chemotherapy in Hodgkin's disease. *Br. Med. J.*, iii, 7.

RAPPAPORT, H. (1966) In *Atlas of Tumour Pathology*, Sect. 3, Fascicle 8. Washington.

ROWE, D. S., CRABBE, P. A. & TURNER, M. W. (1968) Immunoglobulin D in serum, body fluids and lymphoid tissues. *Clin. Exp. Immunol.*, **3**, 477.

SAILER, D., LUTZ, H. & HARTWICH, G. (1973) Quantitativer Immunoglobulinbestimmung (G, A, M) bei Lymphogranulomatoser. *Verh. Dtsch. Ges. Inn. Med.*, **79**, 508.

STEIDLE, C., FATEH-MOGHADAM, A., LAMERZ, R., HUHN, D. & ERHART, H. (1976) Immunoglobuline G, A, M. und E bei Lymphogranulomatose. *Munch. Med. Wschr.*, **118**, 503.

TADA, T. (1975) Regulation of reaginic antibody formation in animals. *Prog. Allergy*, **19**, 122.

WAGENER, D. J. T., VAN MUNSTER, P. J. J. & HAANEN, C. (1976) The immunoglobulins in Hodgkin's disease. *Eur. J. Cancer*, **12**, 683.

WALDMANN, T. A., BULL, J. M., BRUCE, R. M. & 4 others (1974) Serum immunoglobulin E levels in patients with neoplastic disease. *J. Immunol.*, **113**, 379.

WALZER, P. D. & KUNKEL, H. G. (1974) The correlation of serum IgD concentration with Gm allotype. *J. Immunol.*, **113**, 274.